# COVID-19 Vaccine Refusal among Nurses Worldwide: Review of Trends and Predictors

**DOI:** 10.3390/vaccines10020230

**Published:** 2022-02-02

**Authors:** Jagdish Khubchandani, Elizabeth Bustos, Sabrina Chowdhury, Nirbachita Biswas, Teresa Keller

**Affiliations:** College of Health, Education, and Social Transformation, New Mexico State University, Las Cruces, NM 88003, USA; elnleah@nmsu.edu (E.B.); Saabrina@nmsu.edu (S.C.); biswas@nmsu.edu (N.B.); tkeller@nmsu.edu (T.K.)

**Keywords:** nurse, COVID-19, coronavirus, vaccination, vaccine, perception

## Abstract

COVID-19 vaccination hesitancy has become a major concern around the world. Recent reports have also highlighted COVID-19 vaccination hesitancy in healthcare workers. Despite media reports and scientific publications, little is known about the extent and predictors of COVID-19 vaccination refusal among nurses. Thus, the purpose of this study was to assess COVID-19 vaccine refusal rates among nurses globally and to explore the reasons for refusal and factors associated with the uptake of the vaccines. A scoping review of the published literature was conducted, and a final pool of 51 studies (*n* = 41,098 nurses) from 36 countries was included in this review. The overall pooled prevalence rate of COVID-19 vaccine refusal among 41,098 nurses worldwide was 20.7% (95% CI = 16.5–27%). The rates of vaccination refusal were higher from March 2020–December 2020 compared to the rates from January 2021–May 2021. The major reasons for COVID-19 vaccine refusal were concerns about vaccine safety, side effects, and efficacy; misinformation and lack of knowledge; and mistrust in experts, authorities, or pharmaceutical companies. The major factors associated with acceptance of the vaccines were: male sex, older age, and flu vaccination history. Evidence-based strategies should be implemented in healthcare systems worldwide to increase the uptake of COVID-19 vaccines among nurses to ensure their safety and the safety of their patients and community members.

## 1. Background

Nurses are the backbone of modern-day healthcare systems. During the COVID-19 pandemic, nurses played a major role in caring for and treating COVID-19 patients. As a result, many nurses worldwide were infected with COVID-19 and lost their lives [1,2,3,4]. A very conservative estimate by the WHO from summer 2021 suggested that more than 100,000 healthcare workers (HCWs) worldwide (including nurses) died due to COVID-19 infection [4]. Another conservative estimate from the International Council of Nurses suggests that by October 2020, more than 1500 nurses died of COVID-19 across 44 countries, and by January 2021, this number may have exceeded 2200 [5,6]. A recent report from the United States indicated that from March 2020 to April 2021, more than 3600 healthcare workers died due to COVID-19, and almost a third of those who died were nurses [7]. These numbers are estimates and the true representation of COVID-19 related deaths among healthcare workers and nurses is still unknown. While the world is now in the second year of the pandemic, and general life situations may look different for the public in various parts of the world, nurses continue to fight the COVID-19 pandemic at the frontlines, risking their lives and health.

In late 2020 and early 2021, COVID-19 vaccines were rolled out, and in most countries, healthcare workers were designated as priority groups for vaccination [8,9]. There was enormous anticipation for these vaccines, and for healthcare workers, these vaccines were a reason for optimism, a tool to fight the pandemic and continue to serve others while protecting themselves. By late 2021, it was estimated that the majority (>50%) of HCWs (including nurses) in many western countries were either fully vaccinated or planned to get vaccinated [9,10,11]

Despite these optimistic reports from some countries, by summer 2021 vaccine hesitancy and refusal among healthcare workers became a major global concern [8,12,13,14]. For example, the earliest global review conducted in December 2020 included eight studies, and in almost half of the studies, COVID-19 vaccine acceptance rates among healthcare workers (HCWs) were less than 75% [13]. Another review of 76,471 HCWs published in April 2021 estimated that more than a fifth (22.5%) of the HCWs worldwide were hesitant about getting COVID-19 vaccination. Similarly, a review published in May 2021 estimated that COVID-19 vaccine acceptance in HCWs ranged from 27.7% to 77.3% worldwide [14]. These reviews did not specifically provide the proportion of nurses included in their data or the COVID-19 vaccine hesitancy rates exclusively for nurses. Still, and surprisingly, many of these reviews and reports suggest that worldwide, among all HCWs, nurses could have one of the highest hesitancy rates for COVID-19 vaccination [13,14,15,16]. By the last quarter of 2021, some countries around the world started mandating COVID-19 vaccinations for HCWs, including nurses [16,17]. As a result, protests by nurses along with other HCWs garnered substantial media coverage and public discourse. Despite these reports, controversies, and scattered evidence in comprehensive reviews of literature, we could not find a global assessment of COVID-19 vaccination refusal rates among nurses, the reasons for refusal, or the factors associated with COVID-19 vaccination uptake among nurses. Thus, the purpose of this review was to conduct a global assessment of COVID-19 vaccine refusal rates among nurses, reasons for refusal, and factors associated with acceptance (enablers) of vaccination.

## 2. Methods

A scoping review methodology was adopted to synthesize evidence on COVID-19 vaccination refusal rates among nurses by using databases such as PubMed, EBSCO Host, CINAHL, pre-print servers (e.g., medRxiv), and Google Scholar. First, three independent investigators (EB, SC, NB) searched for studies using the following keywords: “nurses”, “nursing”, “coronavirus”, “COVID-19”, “SARS-CoV2”, “vaccine”, “vaccination”, “hesitancy”, “refusal”, “healthcare”, “hospital”, “professional”, “worker”, “employee”. Next, different order of keywords was used by the investigators across repeated searches in scholarly databases to extract the final pool of relevant studies. Then, articles that were to be included in the final review were screened carefully for suitability and to identify other articles that pertained to this review. Additional hand searches were conducted by the senior investigator (JK) to include articles that cited the initially selected pool of articles. Finally, discrepancies on the suitability and relevance of the studies to be included were sorted by discussion (among EV, SAC, NB) with the senior investigators (JK, TK).

**Figure 1:**
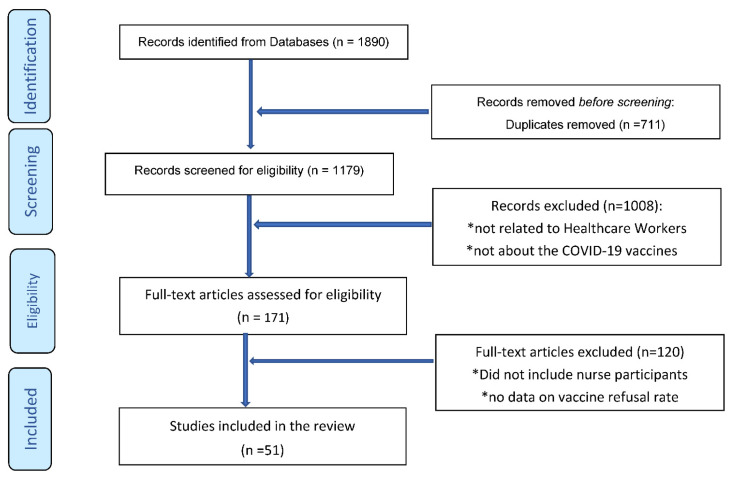
PRISMA Flowchart: Scoping Review of Nurses’ COVID-19 Vaccination Refusal.

The studies that were finally included in this review were those that were published in the English language, between 1 March 2020–30 November 2021, and included nurses as study participants (Figure 1). COVID-19 vaccination refusal rates data were extracted from individual studies if the participating nurses in these studies responded as “no”, “refused”, “declined”, “disagreed”, “did not want to get”, or “do not intend to get” for questions on obtaining the COVID-19 vaccines. Nurses who responded as “unsure”, “uncertain”, and “undecided” about getting COVID-19 vaccines were not grouped with those who refused COVID-19 vaccination. Data were also divided into two categories (March 2020 to December 2020 and January 2021 to October 2021) to explore the difference in global vaccine refusal rates among nurses before (Table 1) and after (Table 2) the COVID-19 vaccines were available for use. Pooled prevalence for COVID-19 vaccination refusal rates was computed from the studies included in this review (with 95% confidence intervals) using random-effects modeling. Subsequently, for each study, the major reasons for COVID-19 vaccine refusal and the enablers of vaccine acceptance were summarized [18,19,20,21,22,23,24,25,26,27,28,29,30,31,32,33,34,35,36,37,38,39,40,41,42,43,44,45,46,47,48,49,50,51,52,53,54,55,56,57,58,59,60,61,62,63,64,65,66,67,68].

## 3. Results

The final pool of articles included 51 studies from 36 countries (Table 1 and Table 2). Multiple studies were conducted in the USA (*n* = 10), China (*n* = 4), France (*n* = 4), Saudi Arabia (*n* = 4), Greece (*n* = 3), and Spain (*n* = 3). Two studies each were found for Cyprus, Germany, Vietnam, Egypt, India, Kuwait, Ghana, Canada, Hong Kong, Palestine, and Turkey; and the rest of the countries had one study each. Females comprised the majority (>50%) of study participants in 48 out of 51 studies. The vast majority of studies (44 of 51) did not exclusively include nurses as participants (i.e., study participants included nurses along with other healthcare professionals). The sample size of participants ranged from 51 to 9701 participating nurses across the studies where data collection occurred between March 2020 and May 2021 [18,19,20,21,22,23,24,25,26,27,28,29,30,31,32,33,34,35,36,37,38,39,40,41,42,43,44,45,46,47,48,49,50,51,52,53,54,55,56,57,58,59,60,61,62,63,64,65,66,67,68].

Before the vaccine rollout (March 2020–December 2020), among the 27 studies with 20,037 nurses from 29 countries, we found that more than a fifth (23.4%) did not want to obtain the COVID-19 vaccines (Table 1) [18,19,20,21,22,23,24,25,26,27,28,29,30,31,32,33,34,35,36,37,38,39,40,41,42,43,44]. In contrast, after the COVID-19 vaccines were available (January 2021–May 2021), among the 24 studies from 18 countries with 21,061 nurses, we found that less than a fifth (18.3%) of the nurses did not want to obtain the vaccine [45,46,47,48,49,50,51,52,53,54,55,56,57,58,59,60,61,62,63,64,65,66,67,68]. Overall, from March 2020 to May 2021, among the 51 studies from 36 countries, for a total sample size of 41,098 nurses, the pooled prevalence rate of COVID-19 vaccine refusal was 20.7% (95% CI = 16.5–27%) [18,19,20,21,22,23,24,25,26,27,28,29,30,31,32,33,34,35,36,37,38,39,40,41,42,43,44,45,46,47,48,49,50,51,52,53,54,55,56,57,58,59,60,61,62,63,64,65,66,67,68].

The major reasons for vaccine refusal were: concerns about COVID-19 vaccine efficacy, safety/speedy approvals, effectiveness, and side effects; mistrust in government/authorities, pharmaceutical companies, and science or experts; misinformation or lack of knowledge about vaccines; and a belief that COVID-19 is a mild disease or does not exist. In contrast, the major factors associated with COVID-19 vaccine acceptance in nurses were: male gender; older age; and history of flu or other vaccinations. Additional factors were higher education/knowledge or work experience; higher perceived risk of getting infected or becoming seriously ill with COVID-19, having chronic diseases; and caring for COVID-19 patients or having greater contact with such patients (Table 1 and Table 2) [18,19,20,21,22,23,24,25,26,27,28,29,30,31,32,33,34,35,36,37,38,39,40,41,42,43,44,45,46,47,48,49,50,51,52,53,54,55,56,57,58,59,60,61,62,63,64,65,66,67,68].

## 4. Discussion

In this global review of COVID-19 vaccination refusal among nurses, we found that more than a fifth (23.4%) refused vaccines in 2020, but slightly less than a fifth (18.3%) refused in 2021. This modest decline may be due to more knowledge and information, popularity, or mass uptake in 2021 (compared to 2020 when the vaccine development and approvals were surrounded by a lot of questions and curiosity). Additionally, it should be noted that most of the studies included in this review were conducted when the vaccines were approved under emergency use authorization. With full approvals and more safety data, vaccine options, and greater availability, the rates of refusal worldwide could decline further. Overall, from March 2020 to May 2021, based on our review, nearly a fifth (20.7%) of the nurses worldwide did not want to obtain COVID-19 vaccination. Additional evidence for a modest decline in COVID-19 vaccine refusal rates among nurses may be seen from previous studies that have found slightly higher rates of vaccine refusal among HCWs or trainees in healthcare professions compared to what we found in our current review [8,12,13,14,15,69]. Despite these optimistic but weak assumptions, the COVID-19 vaccination refusal rates among nurses remain highly disconcerting given the high mortality numbers in nurses, the major frontline role of nurses in caring for COVID-19 patients, and their potential to infect themselves, patients, family, and community members.

Despite the unique working situation and risks faced by nurses, the vaccine refusal rates among nurses remain almost similar to other groups of HCWs and somewhat lower than the general population [11,12,13,14,15,16,34,35,36,37,69,70,71,72,73]. Two recent global reviews found that the rates of COVID-19 vaccine refusal were 22% for college students and 18.9% for trainees/students in healthcare professions [12,71]. A recent global review of 82 studies from 45 countries found the vaccination refusal rates worldwide to be in the range of 0–24% [72]. Similarly, one of the earliest meta-analyses with data until November 2020 found that among 81,173 individuals worldwide, the COVID-19 vaccine refusal/hesitancy rates could be 26.7% [73]. Based on the training, expertise, and work setting of nurses, the refusal rates for COVID-19 vaccines should have been much lower than other groups of individuals who are not HCWs. Continued surveillance and research of predictors, enablers, and barriers to COVID-19 vaccination are warranted to help increase vaccination among nurses.

As a significant proportion of nurses in our review refused to obtain COVID-19 vaccines, we assessed major reasons for refusal and acceptance of vaccination. Surprisingly, the major reasons for COVID-19 vaccination refusal among nurses in this review are very similar to the reasons for refusal observed among other HCWs and the general population (e.g., concerns about vaccine safety, side effects, efficacy; misinformation and lack of knowledge; mistrust in government or pharmaceutical companies and experts) [69,70,71,72,73,74,75,76,77]. Similar to our findings among nurses, studies on other HCWs and the general population have also found that males, older adults, those with flu vaccination history or higher trust in vaccines, and those who perceive themselves as being at higher risk of COVID-19 infections, or believed in protecting others, are more likely to accept the COVID-19 vaccines [12,13,14,15,70,71,72,73,74,75,76,77]. Additionally, the vast majority of study participants in our review were females, and their perspectives are very similar to those of females among the general public (e.g., fear of infertility, getting COVID from the vaccine, serious adverse effects, the influence of social media rumors, misinformation, and conspiracy theories, etc.). While nurses and other HCWs are assumed to have more knowledge about medicine and science, they are not a monolithic group of individuals and are a part of the larger society with multiple external influences on their preferences and perspectives (e.g., family, friends, media, political and social climate, etc.).

Several recommendations and best practices have been suggested to increase uptake of COVID-19 vaccines among nurses, other HCWs, and the general population [66,67,68,69,70,71,72,73,74,75,76,77]. Much of the earlier focus during vaccine roll-out emphasized better communication and education. Recently, mandates, incentives, and workplace policy changes have gained traction in the push to obtain higher vaccination rates. Scholars and experts are also recommending the use of models, such as the five Cs and their extensions. Essentially, to increase COVID-19 vaccine uptake in nurses, healthcare systems should focus on removing Constraints and increasing vaccination Convenience, building Confidence for vaccines and reducing Complacency, as well as using best practices in Communication for unique Context and Cultures [12,13,14,70,71,72,73,74,75,76,77]. Combining this model with additional incentives and mandates might also improve vaccination rates. Nurses play a key role as frontline care providers and are expected to advocate for patient and population health. Increasing the vaccination rate among nurses is an opportunity to improve vaccination rates among other HCWs and the general population (i.e., by role modeling and advocacy).

The findings of this review are subject to several potential limitations [70,71,72,73,74,75,76,77]. First, our data could be limited by the inclusion/exclusion criteria and the search processes we used could have led to the inadvertent omission of certain studies (e.g., only the studies published in English language were included). If so, inherent biases could limit the validity of our findings (e.g., selection bias). Second, vaccine refusal and hesitancy are measured in several different ways across studies (e.g., refused, declined, disagreed, will not take, no, etc.). The lack of uniformity in the measurement of the primary outcome (i.e., vaccine refusal) across studies may have introduced bias in our review. Additionally, if a significant proportion of those who were unsure and undecided about vaccination never get vaccinated, the rates of hesitancy would be much higher than estimates presented in this review. Third, for the studies we included in this review, there were substantial differences in sample sizes, geographic region, and characteristics of study participants, such as age, gender, income, education, health risk profiles, etc. Due to these variations, our estimates of nurses’ vaccine refusal may not appropriately represent COVID-19 vaccination preference for any specific group of nurses. Many studies also had professionals, such as nurse aides, orderlies, midwives, nursing assistants—we did not include these samples in this review, which could limit the generalizability of our findings on COVID-19 vaccination refusal in nursing professions. Finally, we did not adopt a systematic review or meta-analysis methodology because most nurses included in this review were part of a larger sample of HCWs, with few exclusive details on nurse participants reported in the studies included for this review. Given these circumstances, our methods preclude extensive analyses of nurses’ characteristics (e.g., occupational settings or working environments) related to COVID-19 vaccination and robust analysis of quality of studies included for review (e.g., risk of bias analysis). Despite all these limitations, this is one of the largest global assessments of COVID-19 vaccination refusal rates among nurses using rigorous search methods by a team of multiple investigators. Additionally, all the included studies were screened multiple times to assess major reasons for COVID-19 vaccination refusal, and the results were summarized after a consensus among the study team. Finally, our pooled prevalence rates accounted for variations in sample sizes and vaccination refusal rates across studies, providing robust global estimates for COVID-19 vaccination refusal among nurses.

## 5. Conclusions

In this review of COVID-19 vaccine refusal rates among nurses, we found that a little more than a fifth (20.7%) of the nurses worldwide refused to obtain COVID-19 vaccination. The reasons for the refusal of COVID-19 vaccines among nurses are similar to the reasons found in HCWs and, to some extent, in the general public (e.g., concerns about side effects and safety of COVID-19 vaccines). Older individuals, males, and those with past flu vaccination history or compliance with other vaccines were more likely to accept COVID-19 vaccination. Given these findings, healthcare facility leaders and professional nursing organizations should help implement and advocate for measures to increase COVID-19 vaccination uptake among nurses (e.g., educational interventions, incentives, mandates). Researchers and healthcare facility administrators should continue to assess the reasons for COVID-19 vaccination refusal among nurses and help design tailored strategies for reducing vaccine hesitancy among nurses. Nurses have been involved at the frontlines in the battle against COVID-19 and also serve as advocates for population health. To ensure that nurses play a major role in helping vaccinate the general public against COVID-19, COVID-19 vaccination refusal and hesitancy must be reduced among nurses by evidence-based interventions.

## Figures and Tables

**Table 1:** COVID-19 Vaccination Refusal Rates and Reasons Among Nurses (March 2020–December 2020).

Authors and Study Period	Country	Sample Size (*n*)	Refusal Rate (%)	Reasons for Refusal of COVID-19 Vaccines and Enablers of Vaccination
Wang et al. March 2020 [18]	Hong Kong China	806	17.1	Reasons: Concerns about vaccine safety, efficacy, side effects; no perceived need or lack of time Enablers: Male sex, flu vaccination/chronic disease history, contact with COVID-19 patients.
Dror et al. March 2020 [19]	Israel	211 *	39	Reasons: Concerns about vaccine quality, efficacy, side effects; belief COVID-19 is a mild infection. Enablers: Male sex, flu vaccination history, and higher perceived COVID-19 infection risk.
Kwok et al. April 2020 [20]	Hong Kong, China	1205	37	Reasons: Concerns over safety, efficacy, and duration of protection against infection from the vaccine. Enablers: Younger age, collective responsibility belief, and stronger confidence in vaccines.
Nzaji et al. April 2020 [21]	Congo	446 *	76	Reasons: Misinformation and rumors on social media, and use of non-authentic information sources. Enablers: Male sex, older age, occupation type, positive attitude toward the COVID vaccines.
Suo et al. July 2020 [22]	China	3844 *	6.6	Reasons: Concerns about vaccine safety, effectiveness, testing/evaluation process; disease history. Enablers: Male sex, flu vaccination history, higher perceived COVID-19 risk, ED/ER worker.
Gagneux et al. July 2020 [23]	France	371 *	35.3	Reasons: Concerns about vaccine safety, efficacy, effectiveness; and occupational category/work type. Enablers: Male sex, older age, flu vaccination history, and high perceived risk of COVID-19 infection.
Unroe et al. August 2020 [24]	USA	1602 *	60.7	Reasons: Concerns over vaccine side effects/effectiveness/information, religion/politics, lack of trust. Enablers: Male sex, older age, higher perceived risk/severity of COVID-19, higher work experience.
Manning et al. September 2020 [25]	USA	183 *	14.2	Reasons: Concerns over vaccine safety, side effects; low vaccine -related knowledge or trust. Enablers: Male sex, older age, higher perceived risk of COVID-19, willingness to protect others.
Kose et al. September 2020 [26]	Turkey	306 *	13.7	Reasons: Concerns about vaccine efficacy, side effects; low COVID-19 risk, self immunity trust. Enablers: Male sex, young age, flu vaccination history, trust in vaccines or pharma companies.
Baghdadi et al. September 2020 [27]	Saudi Arabia	73 *	41.1	Reasons: Lower perceived COVID-19 risk/severity, lower belief in vaccines, and fear of injections. Enablers: Male sex, middle-aged, lower work experience, and encouragement from family or friends.
AlHassan et al. October 2020 [28]	Ghana	376 *	30.8	Reasons: Concerns about vaccine safety, efficacy, side effects; low vaccine trust, no need for vaccines. Enablers: Female sex, non-Christians/no religious beliefs, higher education, occupational setting.
Verger et al. November 2020 [29]	Canada	1055 *	11.8	Reasons: Concerns over vaccine safety, efficacy; distrust in government; belief in natural immunity Enablers: Male sex, older age, flu vaccination history, a recommendation from others, trust in science.
Barry et al. November 2020 [30]	Saudi Arabia	757 *	49.3	Reasons: Concerns about vaccine side effects, efficacy, speedy development, and lack of safety data. Enablers: Male sex, flu vaccination history, high perceived COVID-19 risk, isolation unit worker.
Grochowska et al. November 2020 [31]	Poland	18 *	27.8	Reasons: Concerns over vaccine safety, efficacy; occupation type, and lower trust in experts/science. Enablers: Male sex, flu vaccination history, occupational status, and recommendation by doctors.
Eguia et al. November 2020 [32]	Spain	51 *	34.6	Reasons: Concerns about vaccine safety, efficacy, side effects; conspiracy theories or misinformation. Enablers: Older age, high general trust in vaccines, never had COVID-19, no chronic disease history.
Ahmed et al. November 2020 [33]	Saudi Arabia	146 *	39.5	Reasons: Concerns over vaccine safety, efficacy; mistrust the company/ country of vaccines/pharma. Enablers: Male sex, older age, chronic disease history, allergies, trust in non-health leaders/other.
Shaw et al. December 2020 [34]	USA	1198 *	25.2	Reasons: Concerns about vaccine safety, side effects, efficacy, speedy development, and lack of data. Enablers: Male sex, older age, higher perceived COVID-19 risk, caring for COVID-19 patients.
Zürcher et al. December 2020 [35]	Switzerland	1690 *	38	Reasons: Concerns about vaccine safety, effectiveness; belief COVID-19 is mild, and PPE is enough. Enablers: Male sex, flu vaccination history, older age, trust in other vaccines and the government.
Fakonti et al. December 2020 [36]	Cyprus, Greece	403 *	40.9	Reasons: Concerns over vaccine side effects, speedy approval, COVID-19 is mild, natural immunity. Enablers: Male sex, older age, flu vaccination history, higher work experience, and private-sector job.
Browne et al. December 2020 [37]	USA	2936 *	12.7	Reasons: Concerns about vaccine side effects, efficacy, information, and mistrust in system/providers. Enablers: Male sex, younger age, higher education/work experience, clinical work setting, white race.
Adeniyi et al. December 2020 [38]	South Africa	591 *	10.8	Reasons: Concerns about vaccine safety, side effects, and lower trust in vaccinations in general. Enablers: Higher education or rank or perceptions of risk; and more contact with COVID-19 patients.
Kaplan et al. December 2020 [39]	Turkey	275 *	33.5	Reasons: Concerns over lack of scientific information, type/choice of vaccine, past COVID-19 infection. Enablers: Male sex, older age, living with family, chronic disease or vaccination compliance history.
Aurilio et al. December 2020 [40]	Italy	531	2.3	Reasons: Concerns over vaccine safety, efficacy, side effects, low perceived COVID-19 severity/risk. Enablers: Male sex, flu vaccination history, younger age, family/friends affected or in bereavement.
Pataka et al. December 2020 [41]	Greece	115 *	13.8	Reasons: Concerns over vaccine safety, side effects; lower education and COVID-19 related knowledge. Enablers: Male sex, older age, married and having children, COVID-19 patient contact/caring for them.
Arora et al. December 2020 [42]	India	53 *	11.4	Reasons: Concerns over vaccine effectiveness duration; prefer other COVID-19 preventive measures. Enablers: Male sex, older age, occupation category, higher education, desire to protect other people.
Chew et al. December 2020 [43]	India, China, Singapore, Indonesia, Bhutan, Vietnam	404 *	3.7	Reasons: Concerns about vaccine safety, effectiveness, side effects, getting COVID from a vaccine. Enablers: Belief that pandemic is very severe, vaccines can help, less internalized stigma about the available vaccines, expressed altruism (or pro-socialness), and high trust in the healthcare authorities.
Aoun et al. December 2020 [44]	Kuwait, Egypt, Saudi, Iraq, Qatar, Syria, Jordan, Bahrain, Lebanon	391 *	16.1	Reasons: Concerns about vaccine side effects, lack of vaccine information, and living in crowded places. Enablers: Male sex, flu vaccination history, chronic disease history, and a desire for the COVID-19 vaccination to protect family/friends.
Total = 27 studies	29 Countries	20,037 Nurses	23.4% (95%CI = 17.1–31.5)	Top Refusal Reasons: Concerns about COVID-19 vaccine safety, side effects, effectiveness. Top Enablers of COVID-19 Vaccination: Male sex, flu vaccination history, older age.

* indicates that the nurse participants were part of a larger sample of HCWs and the reasons and enablers listed are for the whole sample. Data collection months for each study have been arranged in chronological order in the table. All the studies listed in this table had females as the majority (>50%) of study participants.

**Table 2:** COVID-19 Vaccination Refusal Rates and Reasons Among Nurses (January 2021–May 2021).

Authors and Study Period	Country	Sample Size (*n*)	Refusal Rate (%)	Reasons for Refusal of COVID-19 Vaccines and Enablers of Vaccination
Rabi et al. January 2021 [45]	Palestine	639	18	Reasons: Concerns about vaccine side effects; natural immunity preference; media misinformation. Enablers: Older age, no fear of injections; believing in vaccine efficacy; take to protect others.
Hara et. al. January 2021 [46]	Japan	369 *	18.4	Reasons: Concerns about vaccine side effects, effectiveness, newness, vaccine immunity duration. Enablers: Male sex, flu vaccination history, older age, current smoking, local area epidemic situation.
Fares et al. January 2021 [47]	Egypt	89 *	32.6	Reasons: Concerns over vaccine safety, side effects, efficacy; low trust or a lack of information. Enablers: Male sex, COVID-19 patient care, past vaccination history, recommendations from others.
Spinewine et al. January 2021 [48]	Belgium	319 *	5.3	Reasons: Concerns over vaccine side effects and efficacy on variants, low perceived COVID-19 risk. Enablers: Male sex, older age, flu vaccination history, to protect others and to stop the pandemic.
Maraqa et al. January 2021 [49]	Palestine	483 *	75.6	Reasons: Concerns over vaccine side effects, efficacy, could get COVID-19 infection from a vaccine. Enablers: Male sex, flu vaccination history, younger age, higher knowledge/risk of COVID.
Desveaux et al. January 2021 [50]	Canada	1556 *	18.8	Reasons: Concerns about vaccine safety/speedy approval; belief that vaccines are it is not required, lower confidence in vaccines. Enablers: Older age, higher education, trust in public health websites/providers, paid sick days at job.
Mena et al. January 2021 [51]	Spain	234 *	12.8	Reasons: Concerns about vaccine effectiveness; lower education and income; COVID-19 patient care. Enablers: Male sex, flu vaccination history, older age, and high perceived risk of COVID-19 infection.
Schrading et al. January 2021 [52]	USA	345 *	22.3	Reasons: Concern over vaccine safety, efficacy, religious/ethical/personal reason, and lack of time Enablers: Male sex, older age, white race, chronic disease history, and easier logistics to get vaccines.
Patelarou et al. January 2021 [53]	Albania, Cyprus, Spain, Greece, Kosovo	1135	4.8	Reasons: Concerns about vaccine safety, efficacy, effectiveness; previous COVID-19 infection, lower perceived risk or severity of COVID-19 infection. Enablers: Male sex, flu vaccination history, greater knowledge of COVID-19 vaccines, trust in experts and the government, and living in an area of high mortality from COVID-19 infections.
Holzmann-Littig et al. February 2021 [54]	Germany	466 *	9	Reasons: Concerns over vaccine side effects, safety; lack of trust in authorities/pharma companies. Enablers: Older age, trust in other vaccines, high perceived risk or knowledge about COVID-19.
Baniak et al. February 2021 [55]	USA	276	5.1	Reasons: Concerns about vaccine side effects, efficacy; and lack of information to make a decision. Enablers: Confidence in vaccine safety, development process, and higher work experience years.
AlKetbi et al. February 2021 [56]	UAE	1402 *	11.3	Reasons: Concern about vaccine side effects, lack of information, belief the vaccines may not work. Enablers: Male sex, older age, trust in vaccine producers/distributors, belief vaccines are effective.
Agyekum et al. February 2021 [57]	Ghana	151 *	64.9	Reasons: Concerns about vaccine safety, side effects; had COVID-19 or low perceived risk of disease. Enablers: Male sex, occupation type, family/friends had COVID-19, and higher trust in government.
Huynh et al. Feb 2021 [58]	Vietnam	146 *	16.4	Reason: Concerns about vaccine efficacy, side effects, fear, doubt; low perceived vaccine benefits. Enablers: High perceived risk or knowledge about COVID-19; reliable sources of information.
Paris et al. February 2021 [59]	France ^β^	563 *	3	Reasons: Concerns about vaccine safety, efficacy; controversies about the existing vaccine side effects. Enablers: Older age, flu vaccination history, no comorbidities or allergies, past COVID-19 infection.
Janssen et al. March 2021 [60]	France	821 *	15.5	Reasons: Concerns about vaccine side effects, efficacy, immunity duration; mistrust in pharma group Enablers: Male sex, older age, flu vaccine or chronic disease history, higher COVID-19 infection risk
Angelo et al. March 2021 [61]	Ethiopia ^β^	242 *	55.4	Reasons: Concerns about vaccine side effects; misinformation; negative attitude toward prevention. Enablers: Chronic disease history; high knowledge of COVID-19/ vaccines, higher COVID-19 risk.
Al-Sanafi et al. March 2021 [62]	Kuwait	127 *	17.3	Reasons: Concerns about vaccine side effects, influence of conspiracy theories or social media stories. Enablers: Male sex, higher education and COVID-19 vaccine confidence, job in public health sector.
Vignier et al. March 2021 [63]	France	200 *	35.5	Reasons: Concerns about vaccine side effects, benefits, efficacy, and low trust in pharma/authorities. Enablers: Male sex, older age, vaccination history, COVID-19 patient care, higher COVID-19 risk.
Branson et al. April 2021 [64]	USA	311	12.2	Reasons: Concerns over vaccine safety, side effects, speedy approval, previous COVID diagnosis. Enablers: Older age, white race, higher education/work experience/designation or perceived risk.
Kozak et al. April 2021 [65]	Germany	908 *	9.9	Reasons: Concerns about vaccine safety/efficacy, side effects/fertility; low confidence/social pressure. Enablers: Middle age, flu vaccination history, COVID-19 patient contact, desire to protect others.
Do et al. April 2021 [66]	USA	275 *	47.6	Reasons: Concerns about vaccine side effects, efficacy, newness; mistrust; past COVID-19 diagnosis. Enablers: Male sex, older age, flu vaccination history, chronic disease history, COVID-19 patient care.
Toth-M, et al. May 2021 [67]	USA	303 *	20.5	Reasons: Concerns over lack of information/evidence on vaccines, side effects; racial minorities. Enablers: Male sex, older age, liberal, chronic disease in the family, recommendation of others.
King et al. May 2021 [68]	USA ^β^	9701 *	11.6	Reasons: Concerns about vaccine side effects, efficacy, vaccine not needed, low trust in government. Enablers: Favorable attitudes toward vaccines/vaccination overall, and recommendation from others.
Total = 24 studies	18 Countries	21,061 Nurses	18.3% (95%CI = 14.4–24.7)	Top Refusal Reasons: Concerns about vaccine side effects, efficacy, and misinformation/mistrust. Top Enablers of COVID-19 Vaccination: Male sex, flu vaccination history, older age.
**Grand Total** = Tables 1 + 2 = 51 Studies, 36 Countries, 41,098 Nurses. COVID-19 vaccine refusal rate from March 2020–May 2021 = 20.7% (95% CI = 16.5–27.0)				

∗ indicates that the nurse participants were part of a larger sample of HCWs and the reasons and enablers listed are for the whole sample. Data collection months for each study have been arranged in chronological order in the table. β indicates studies without the majority of the study participants being females (rest had a majority of females). The overall prevalence of vaccine refusal among nurses was estimated from the included studies with 95% confidence intervals using random-effects modeling.

## Data Availability

All data is available within the studies included for this review.
